# Acute and late toxicity patterns of moderate hypo-fractionated radiotherapy for prostate cancer: A systematic review and meta-analysis

**DOI:** 10.1016/j.ctro.2023.100612

**Published:** 2023-03-17

**Authors:** F. Sinzabakira, V. Brand, W.D. Heemsbergen, L. Incrocci

**Affiliations:** a Department of Radiotherapy, Erasmus MC Cancer Institute, Dr Molewaterplein 40, 3015 GD Rotterdam, The Netherlands; b Department of Clinical Oncology, Rwanda Military Hospital, Street KK739ST, Kicukiro District, Kigali City, Rwanda

**Keywords:** Prostate cancer, Radiotherapy, Hypofractionation, Toxicity

## Abstract

**Introduction:**

Moderate hypofractionated (HF) radiotherapy is becoming the new standard in radiotherapy for prostate cancer patients. It is established as safe, but it might be associated with increased acute toxicity levels. We conducted a systematic review on moderate HF to establish acute toxicity levels and their required clinical management; late toxicity was reported as a secondary outcome.

**Material and methods:**

Using PRISMA guidelines, we conducted a systematic review for studies published until June 2022. We identified 17 prospective studies, with 7796 localised prostate cancer patients, reporting acute toxicity of moderate hypofractionation (2.5–3.4 Gy/fraction). A meta-analysis was done for 10/17 studies with a control arm (standard fractionation (SF)), including evaluation of late toxicity rates. We used Cochrane bias assessment and Newcastle-Ottawa bias assessment tools for randomized controlled trials (RCTs) RCT and non-RCTs, respectively.

**Results:**

Pooled results showed that acute grade ≥ 2 gastro-intestinal (GI) toxicity was increased by 6.3 % (95 % CI for risk difference = 2.0 %–10.6 %) for HF vs SF. Acute grade ≥ 2 Genito-urinary (GU) and late toxicity were not significantly increased. The overall risk of bias assessment revealed a low risk in the meta-analysis of included studies. Data on management of toxicity (medication, interventions) was only reported in 2/17 studies.

**Conclusion:**

HF is associated with increased acute GI symptoms, needing adequate monitoring and management. Reports on toxicity management were very limited. Pooled late GI and GU toxicity showed similar levels for SF and HF.

## Highlights

• Moderate hypofractionated (HF) radiotherapy is becoming the new standard in radiotherapy for prostate cancer patients. It is established as safe, but it might be associated with increased acute toxicity levels.
• Using PRISMA guidelines, we conducted a systematic review for studies published until June 2022. We identified 17 prospective studies reporting acute toxicity of moderate hypofractionation (2.5–3.4 Gy/fraction). A meta-analysis was done for 10/17 studies with a control arm. We used Cochrane bias assessment and Newcastle-Ottawa bias assessment tools for randomized controlled trials (RCTs) RCT and non-RCTs, respectively.
• Pooled results showed that acute grade ≥ 2 gastro-intestinal (GI) toxicity was relatively increased by 6.3 % (95 % CI = 2.0 %–10.6 %) in HF group, acute grade ≥ 2 Genito-urinary (GU) in HF was not significantly increased. The overall risk of bias assessment revealed a low risk in the meta-analysis of included studies.
• Available data suggest increased GI symptoms in the acute phase, needing adequate monitoring and management.

## Introduction

Radiotherapy dose is traditionally delivered in fractions of 1.8–2 Gy per fraction, with the main purpose to spare normal tissues without compromising tumor control. With recent technological developments in radiotherapy, highly conformal dose delivery offers the possibility to safely deliver fractions of more than 2 Gy while sparing adjacent healthy tissue [1].

Recent randomized clinical trials (RCTs) have shown that moderate hypofractionation (HF) in prostate cancer treatment is effective and safe [2], [3], [4], [5], [6]. Especially the dose-fractionation schedules of 60 Gy in 20 fractions of 3 Gy, and 62 Gy in 20 fractions of 3.1 Gy are of interest [4], [5], [7]. Compared to the previous standard fractionation of 74–78 Gy in 2 Gy fractions, the number of fractions and number of treatment days decrease, which will increase patient convenience, and reduce the linear accelerator time by almost 50 %. As a result, costs are reduced, and in a situation where the availability of a linear accelerator is limited more patients will have a chance to be treated.

The limited radiation therapy services for cancer control worldwide have triggered a big interest in developing affordable and time saving radiotherapy techniques to increase access to those resources. Hypofractionated radiotherapy regimens could reduce the treatment cost and increase accessibility in countries with limited resources. According to a growing body of evidence, hypofractionation should be the most advised technique to overcome global shortage of radiotherapy resources [8], [9], [10], [11], [12]. However, radiation also causes acute tissue damage that resolves over time, but still can be problematic during the acute phase of the radiotherapy, i.e. at the end of treatment and the weeks thereafter. HF seems to increase this acute response, especially with respect to gastrointestinal (GI) and genitourinary (GU) complaints as reported by several studies [2], [4], [6], [13]. Therefore, there is a need to study further the acute phase of hypofractionated radiotherapy to gain a better understanding of GI and GU toxicity profiles. Concerning late toxicity, several review studies concluded that late toxicity rates are not increased with moderate HF [14], [15], [16], [17].

The primary objective of this study is to establish acute toxicity levels in moderate HF (2.4–3.4 Gy/fraction) and the required clinical management for prostate cancer patients. Late toxicity rates will be reported as secondary outcome.

## Material and methods

For this study, we applied the Preferred Reporting Items for Systematic Reviews and meta-analysis (PRISMA) guidelines [18]. The full document is located in appendix section (Appendix B).

### Selection criteria

The selection of studies to be included was done according to inclusion criteria for the literature search (PICOS) [19] that is summarized below:

(1) Population: Men with pathologically proven adenocarcinoma of the prostate, low-, intermediate and high-risk localized disease.
(2) Intervention: Studies that enrolled patients for external beam radiotherapy (EBRT), moderate HF schedules with curative intent were included. These schedules were recommended by ASTRO (American Society for Radiation Oncology), ASCO (American Society of Clinical Oncology) and AUA (American Urological Association) in evidence-based guidelines to be safe and effective in low, intermediate, and high-risk diseases [20]. In this document, moderate HF is defined as fraction size 2.4–3.4 Gy and ultra-HF as fraction size ≥ 5 Gy.
(3) Comparator: A Control group was not considered mandatory because the focus was on the toxicity profiles and therapeutic interventions and not on the comparison with standard fractionation.
(4) Output: Acute and late grade ≥ 2 GI and GU toxicity as reported in every study according to all toxicity reporting systems such as EORTC (European Organization for research and Treatment of Cancer), RTOG (Radiation Therapy Oncology Group), CTCAE (Common Terminology Criteria for Adverse Effect) all versions and LENT-SOMA (Late effects Normal Tissue Task Force – Subjective, Objective, Analytics) scales.
(5) Study type: We have included prospective phase II and III studies that were published in English between January 2010 and June 2022.

### Information sources

The search was performed using Medline (PubMed), Embase, Science direct, and the Cochrane library data bases.

### Search strategy

The search strategy used in PubMed using was: ((“prostatic neoplasms”[MeSH Terms] OR (“prostatic”[All Fields] AND “neoplasms”[All Fields]) OR “prostatic neoplasms”[All Fields] OR (“prostate”[All Fields] AND “cancer”[All Fields]) OR “prostate cancer”[All Fields]) AND (“radiotherapy”[MeSH Terms] OR “radiotherapy”[All Fields] OR “radiotherapies”[All Fields] OR “radiotherapy”[MeSH Subheading] OR “radiotherapy s”[All Fields]) AND (“hypofractionated”[All Fields] OR “hypofractionation”[All Fields]) AND (“toxic”[All Fields] OR “toxical”[All Fields] OR “toxically”[All Fields] OR “toxicant”[All Fields] OR “toxicant s”[All Fields] OR “toxicants”[All Fields] OR “toxicated”[All Fields] OR “toxication”[All Fields] OR “toxicities”[All Fields] OR “toxicity”[MeSH Subheading] OR “toxicity”[All Fields] OR “toxicity s”[All Fields] OR “toxics”[All Fields])) AND (2010:2022[pdat]). The publications found were categorized according to Titles/abstract/full text and reviewed by 2 authors (FS and VB). After crosschecking, all reviewed referenced articles were screened for relevant information.

### Data selection process

Extraction of data for each study was independently performed by 2 authors (**FS** and **VB**) using the PRISMA statement updated guideline (Appendix B). All data were verified by the senior author (**WH**) and any raised discrepancy was resolved by group discussion.

### Data items

For each selected publication we collected baseline information such as author, year of publication, country, setting, trial phase, interventional model, sample size (baseline and endpoint), risk stratification, radiotherapy treatment technique, target volume, dosimetry and androgen deprivation therapy (ADT) use (Table 1). For the endpoints of interest we collected available data on acute and late GI and GU toxicity rates, the scoring criteria used, and their management procedures. (Table 2 and Table 3).

**Table 1:** Characteristics of included studies.

Author, year, ref, country	Study phase	Study design	Interventional model	Baseline sample/ Endpoint sample	Risk group (L,I,H) %	Technique	Target volume	Total dose (Gy)/n fx/daily fx	ADT (%)
Aluwini et al., 2015, 2016 [2], [6]	Phase III	Randomized, multicentred	Parallel groups	410/403	I: 27, H:73	IMRT	Prostate + SV	HF: 64.6/19 × 3.4	66
Netherlands				410/391				SF: 78/39 × 2	67
Dearnaley et al., 2016 [4]	Phase III	Randomized, multicentred	Parallel groups	1074 /720	L:15, I:73, H:12	IMRT	Prostate + (−SV)	HF: 60/20 × 3	97
(UK)				1077 /713				HF: 57/20 × 3	97
				1065 /715				SF: 74/37 × 2	96
Arcangeli et al., 2011 [7]	Phase III	Randomized, single centred	Parallel groups	83/83	H:100	3DCRT	Prostate + SV	HF: 62/20 × 3.1	100
Italy				85/85				SF: 80/40 × 2	100
Catton et al., 2017 [5]	Phase III	Randomized, multicentred	Parallel groups	608/601	I:100	IG-IMRT	Prostate + SV	HF: 60/20 × 3	0
Canada				598/591				SF: 78/39 × 2	0
Lee et al., 2016 [3]	Phase III	Randomized, multicentred	Parallel groups	550/545	L:100	3DCRT/IMRT	Prostate	HF: 70/28 × 2.5	0
(USA)				542/534				SF: 73.8/41 × 1.8	0
Karklelyte et al., 2018 [21]	Phase II	Randomised, single centred	Parallel groups	115/115	H:100	IG-IMRT-SIB	Prostate + SV	HF: 63/20 × 3.15	100
Lithuania				106/106				SF: 76/38 × 2	100
Viani et al., 2013 [22]	Phase III	Randomized, single centre	Parallel groups	112/112	L:33.6, I:38.7, H:32.2	3DCRT	Prostate+(−SV)	HF: 69/23 × 3	62.5
Brazil				105/105				SF: 78/39 × 2	80
Norkus et al., 2013 [23]	Phase III	Randomized, single centre	Parallel groups	57/53	H:100	IG-IMRT	Prostate + SV	HF: 63/20 × 3.15	100
Lithuania				67/59				SF: 76/38 × 2	100
Mc Donald et al., 2013 [24]	Phase II	Observational, single centre	Parallel groups	75/75	H:100	IMRT	Prostate + SV	HF: 70/28 × 2.5	93
USA				82/82				SF: 75–77/(1.8–2)	91
Kozuka et al., 2017 [25]	Phase II	Observational, single centre	Parallel groups	31/31	I:100	IMRT	Prostate + SV	HF: 70/28 × 2.5	41.9
Japan				86/86				SF: 78/39 × 2	52.3
Krupa et al., 2016 [26]Czech Republic	Phase II	Observational, single centre	Single arm	158/158	L:30, I: 59, H:10	VMAT	Prostat+(−SV)	HF: 60/20 × 3	54
Faria et al., 2017 [27]Canada	Phase II	Observational, single centre	Single arm	105/105	H:100	IMRT-SIB	Prostate + SV	HF: 60/20 × 3	100
Tramacere et al., 2015 [28]Italy	Phase II	Observational, single centre	Single arm	97/97	L:19, I:41, H:40	3DCRT	Prostate+(−SV)	HF:62/20 × 3.1	100
Valeriani et al., 2014 [29]Italy	phase II	Observational, single centre	Single arm	82/82	H:100	IGRT/IMRT	Prostate + SV	HF: 68.75/25 × 2.75	100
Lock et al., 2010 [30]Canada	Phase II	Observational, single centred	Single arm	66/66	L:40.9, I:54.5, H:4.5	IMRT	Prostate+(−SV)	63.2/20 × 3.16	9.1
Pervez et al., 2010 [31]Canada	Phase II	Observational, single centre	Sigle arm	60/60	H:100	IMRT	Prostate + SV	68/25 × 2.72	100
White et al., 2015 [32]UK	Phase II	Observational, single centre	Single arm	90/90	L:11, I:38, H:51	3DCRT	Prostate +(−SV)	57/19 × 3	71

Abbreviations: SF (Standard Fractionation); HF(Hypofractionation); VMAT (Volumetric Modulated Arc Therapy); IMRT (Intensity Modulated Radiation Therapy); IGRT (Image Guided Radiation Therapy); SIB (Simultaneous Integrated Boost), Gy (Gray), 3DCRT (Three Dimensional Conformal Radiation Therapy),ADT((Androgen Deprivation Therapy); L: Low risk, I: Intermediate risk; H: High risk; SV: Seminal vesicles; UK: United Kingdom; USA: United States of America.

**Table 2:** Acute and late Gastrointestinal (GI) toxicity patterns.

Study	Arm	BED (α/β = 10)	Acute G ≥ 2 GI toxicity	Late G ≥ 2 GI toxicity	Scoring system	Toxicity management
Aluwini et al.	HF: 64.6/19 × 3.4	86.56 Gy	169/403	87/395	EORTC-RTOG	NR
	SF: 78/39 × 2	93.60 Gy	122/391	66/387		
Dearnaley et al.	HF: 60/20 × 3	78.00 Gy	277/720	28/959	EORT-RTOG,LENT-SOMA	NR
	HF: 57/20 × 3	74.10 Gy	270/713	17/962		
	SF: 74/37 × 2	88.80 Gy	176/715	35/922		
Arcangeli et al.	HF: 62/20 × 3.1	81.22 Gy	29/83	12/83	EORTC-RTOG, LENT-SOMA	NR
	SF: 80/40 × 2	96.00 Gy	18/85	10/85		
Catton et al.	HF: 60/20 × 3	78.00 Gy	95/608	45/608	EORT-RTOG	NR
	SF: 78/39 × 2	93.60 Gy	59/598	66/598		
Karklelyte et al.	HF: 63/20 × 3.15	82.84 Gy	55/115	NR	EORTC-RTOG	NR
	SF: 76/38 × 2	91.20 Gy	40/106	NR		
Viani et al.	HF: 69/23 × 3	89.70 Gy	21/112	NR	EORTC-RTOG	antispasmodics, analgesics (n? unknown)
	SF: 78/39 × 2	93.60 Gy	18/105	NR		
Norkus et al.	HF: 63/20 × 3.15	82.84 Gy	8/59	NR	EORTC-RTOG	NR
	SF: 76/38 × 2	91.20 Gy	7/53	NR		
Krupa et al.	HF: 60/20 × 3	78.00 Gy	37/158	NR	EORTC-RTOG	NR
Tramacere et al.	HF: 62/20 × 3.1	81.22 Gy	15/97	8/97	EORTC-RTOG	NR
Valeriani et al.	HF: 68.5/25 × 2.7	87.66 Gy	4/59	NR	EORTC-RTOG	NR
Pervez et al.	HF: 68/25/2.72	86.50 Gy	21/60	NR	EORTC-RTOG	NR
Lee et al.	HF: 70/28 × 2.5	87.50 Gy	54/545	99/545	CTCAE, LENT-SOMA	NR
	SF: 73.8/41 × 1.8	78.08 Gy	52/534	61/534		
Kozuka et al.	HF: 70/28 × 2.5	87.50 Gy	6/31	1/31	CTCAE	NR
	SF: 78/39 × 2	93.60 Gy	14/86	3/86		
Mc Donald et al.	HF: 70/28 × 2.5	87.50 Gy	27/75	10/75	CTCAE, LENT-SOMA	Endoscopic coagulation, blood transfusion (n = 1)
	SF: 75–77/(1.8–2)	88.50 Gy; 92.40 Gy	29/82	20/82		
Faria et al.	HF: 60/20 × 3	78.00 Gy	17/105	7/105	CTCAE	NR
Lock et al.	HF: 63.2/20 × 3.16	83.17 Gy	22/66	16/66	CTCAE	NR
White et al.	HF: 57/19 × 3	74.10 Gy	8/90	8/90	CTCAE	NR

Abbreviations: SF (Standard Fractionation); HF(Hypofractionation); RTOG (Radiation Therapy Oncology Group), CTCAE (Common Terminology for Adverse Events); EORTC (European Organisation for Research and Treatments of Cancer), LENT-SOMA (Late effects Normal Tissue Task Force)-(Subjective, Objective, Management, Analytic scales; NR (Not Reported).

**Table 3:** Acute and late Genitourinary (GU) toxicity patterns.

Study	Arm	BED(α/β = 10)	Acute G ≥ 2 GU toxicity	Late G ≥ 2 GU toxicity	Scoring system	Acute toxicity management
Aluwini et al.	HF: 64.6/19 × 3.4	86.56 Gy	244/403	163/395	EORTC-RTOG	NR
	SF: 78/39 × 2	93.60 Gy	226/391	151/387		
Dearnaley et al.	HF: 60/20 × 3	78.00 Gy	365/720	16/959	EORT-RTOG, LENT-SOMA	NR
	HF: 57/20 × 3	75.10 Gy	327/713	11/962		
	SF: 74/37 × 2	88.80 Gy	327/713	12/922		
Arcangeli et al.	HF: 62/20 × 3.1	81.22 Gy	39/83	7/83	EORTC-RTOG,LENT-SOMA	NR
	SF: 80/40 × 2	96.00 Gy	34/85	5/85		
Catton et al.	HF: 60/20 × 3	78.00 Gy	161/608	123/608	EORT-RTOG	NR
	SF: 78/39 × 2	93.84 Gy	159/598	116/598		
Karklelyte et al.	HF: 63/20 × 3.15	82.84 Gy	31/115	NR	EORTC-RTOG	NR
	SF: 76/38 × 2	91.20 Gy	30/106	NR		
Viani et al.	HF: 69/23 × 3	89.70 Gy	21/112	NR	EORTC-RTOG	alpha blokker, analgesics (n?)
	SF: 78/39 × 2	93.60 Gy	18/105	NR		
Norkus et al.	HF: 63/20 × 3.15	82.84 Gy	26/59	NR	EORTC-RTOG	NR
	SF: 76/38 × 2	91.20 Gy	23/53	NR		
Krupa	HF: 60/20 × 3	78.00 Gy	22/158	NR	EORT-RTOG	NR
Tramacere et al.	HF: 62/20 × 3.1	81.22 Gy	23/97	11/97	EORT-RTOG	NR
Valeriani et al.	HF: 68.5/25 × 2.7	87.66 Gy	2/59	NR	EORT-RTOG	NR
Pervez et al.	HF: 68/25/2.72	86.50 Gy	20/30	NR	EORT-RTOG	NR
Lee et al.	HF: 70/28 × 2.5	87.50 Gy	129/545	142/545	CTCAE	NR
	SF: 73.8/41 × 1.8	78.08 Gy	132/534	109/534		
Kozuka et al.	HF: 70/28 × 2.5	87.50 Gy	15/31	5/31	CTCAE	NR
	SF: 78/39 × 2	93.60 Gy	44/86	15/86		
Mc Donald et al.	HF: 70/28 × 2.5	87.50 Gy	33/75	5/75	CTCAE	NR
	SF: 75–77/(1.8–2)	88.50 Gy; 92.40 Gy	40/82	3/82		
Faria et al.	HF: 60/20 × 3	78.00 Gy	19/105	8/105	CTCAE	NR
Lock et al.	HF: 63.2/20 × 3.16	83.17 Gy	22/66	9/66	CTCAE	NR
White et al.	HF: 57/19 × 3	57/19 × 3	9/90	2/90	CTCAE	NR

Abbreviations: SF (Standard Fractionation); HF(Hypofractionation); RTOG (Radiation Therapy Oncology Group), CTCAE (Common Terminology for Adverse Events); EORTC (European Organisation for Research and Treatments of Cancer); LENT-SOMA (Late effects Normal Tissue Task Force)-(Subjective, Objective, Management, Analytic scales; NR (Not Reported).

### Risk of bias assessment

Risk of bias for selected papers was independently assessed by 2 authors (FS and VB). For RCT we applied the Cochrane risk of bias tool that categorise bias as low, unclear (some concerns) and high risk (Appendix C). For observational comparative trials, we used Newcastle-Ottawa risk of bias tool that was adapted graphically and converted into 2 stars, 1 star and 0 representing low, unclear, and high risk respectively) (Appendix D).

### Outcomes effect measures

Our primary end point was to establish acute toxicity levels of prostate cancer patients treated with moderate HF (2.4–3.4 Gy/fraction) by reporting acute GI and GU toxicity outcomes in proportions. Moreover, to establish the increase compared to previous SF, we calculated their proportion differences presented by risk difference and corresponding 95 % Confidence Interval (CI). Late toxicity rates were reported as a secondary outcome.

### Data synthesis methods

We tabulated selected studies characteristics (author, year of publication, country, setting, trial phase, interventional model, sample (baseline and endpoint), patients characteristics and cancer patterns (age, risk group, radiotherapy technique, target volume, dosimetry, Biological Effective Dose (BED) and ADT use in Table 1. Proportions of acute and late GU and GI adverse events, and their clinical management are reported in Table 2 and Table 3. Studies with single arms were excluded from meta-analysis. Then, for each study with comparison groups (HF vs SF), the specific RD in proportions of individuals who had acute/late grade ≥ 2 GI and GU toxicity between HF schedules and SF and their corresponding 95 % CI were pooled into a summary of RD by Mantel-Haenszel method (Table 4, Table 5) supplementary file). We constructed forest plots whose diamond located at the bottom represents a summary of the best estimate RD meta-analysis results and its width stands for corresponding 95 % CI (Fig. 2, Fig. 3 for acute toxicity and Fig. 4, Fig. 5 for late toxicity patterns). For the meta-analysis we used StatsDirect software (StatsDirect ltd Wirral, UK Company number: 04399867) with a p value ≤0.05 considered statistically significant. Heterogeneity between studies was evaluated by Cochran Q test and its magnitude was assessed by I^2^ test that measures the percentages of variability caused by actual heterogeneity rather than chance. It is represented by different values with <25 %, 25 %–50 % and ˃50 % denoting minimal, moderate, and substantial heterogeneity respectively. We used random effect models for substantial heterogeneity and fixed effect models for less heterogeneity. We used subgroup analysis to search for specific study characteristics that could cause substantial heterogeneity and analyze its impact on the pooled estimate.

**Fig. 1:**
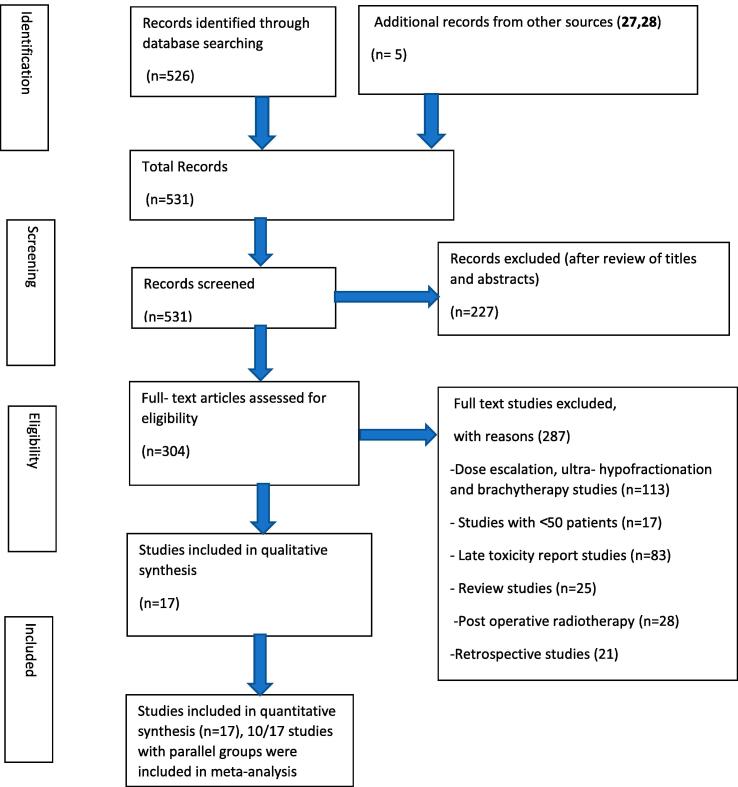
PRISMA flow diagram- Study selection.

**Fig. 2:**
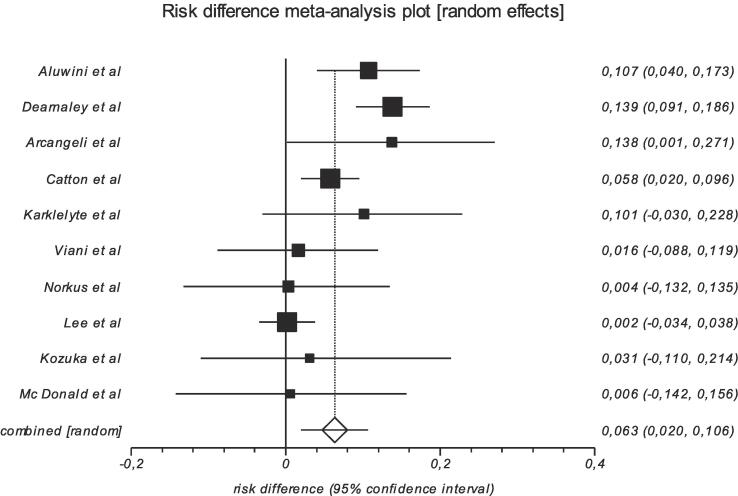
Pooled analysis of risk of worse acute GI toxicity after moderate HF vs SF radiotherapy.

**Fig. 3:**
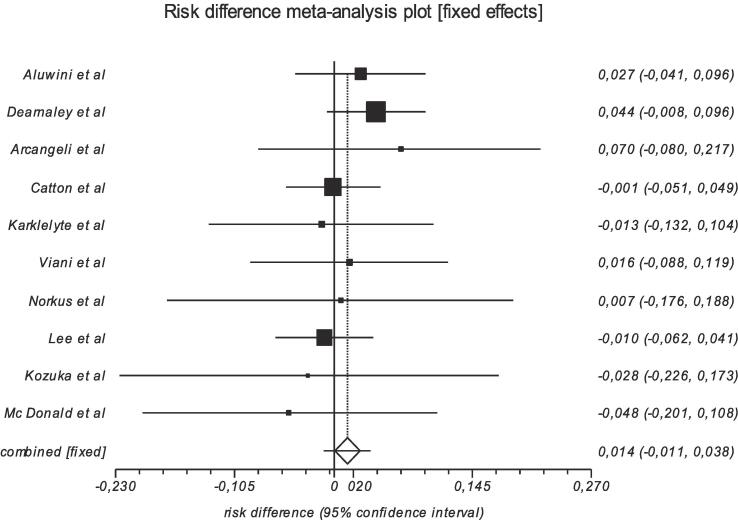
Pooled analysis of risk of worse acute GU toxicity after moderate HF vs SF radiotherapy.

**Fig. 4:**
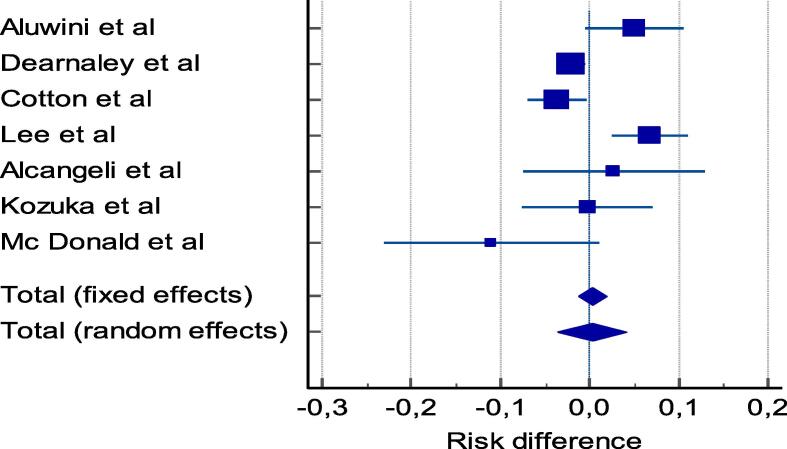
Pooled analysis of risk of late GI toxicity after moderate HF vs SF radiotherapy.

**Fig. 5:**
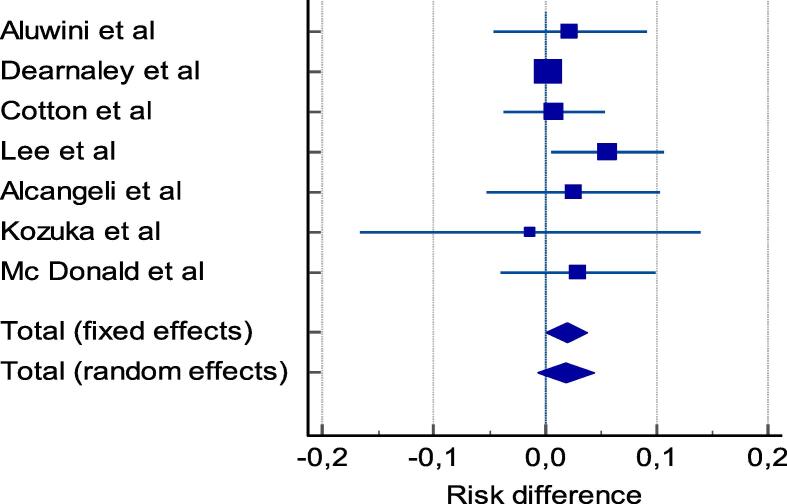
Pooled analysis of risk of late GU toxicity after moderate HF vs SF radiotherapy.

## Results

### Study selection

The literature search resulted in 531 unique records of which 227 publications were excluded after the review of titles and abstracts. From the remaining 304 articles which were assessed for eligibility, 287 studies were excluded with reasons mentioned in Fig. 1. The flow diagram for study selection is depicted in Fig. 1. A total of 17 studies were included [7], [2], [3], [4], [5], [21], [22], [23], [24], [25], [26], [27], [28], [28], [29], [30], [31], of which 10 studies had control arms and could therefore be included in the meta-analysis [7], [2], [3], [4], [5], [21], [22], [23], [24], [25]. The selected studies characteristics according to PICOS criteria are summarized in Table 1 and the characteristics related to acute/late GI and GU adverse events and their management procedures are summarized in Table 2 and Table 3 respectively.

### Study characteristics

All involved studies were published between year 2011 and 2022. We included 10 prospective phase II studies and 7 Phase III RCTs. Among 17 included studies; 7 were single arm and 10 with parallel groups comparing SF and moderate HF. These studies included 7796 treated patients (Table 1).

### Patient characteristics and selection criteria

All studies treated men with localized prostate cancer, aged between 44 and 88 years, and a median age ≈70 years. In all studies, patients with localized prostate cancer were included; patients with previous pelvic irradiation, previous radical prostatectomy, distant metastasis, and poor performance status were excluded.

### Tumour and treatment characteristics

The target volume included prostate only for low-risk, and prostate plus (part of) the seminal vesicles for intermediate and high-risk patients. Most patients received ADT before and/or during radiotherapy except 2 studies where men did not received ADT [7], [9]. In addition, 4 studies treated patients with 3DCRT (3-Dimensional Conformal Radiation Therapy) only, 12 studies treated patients with IMRT/VMAT (Intensity modulated Radiotherapy/Volumetric Modulated Radiotherapy), and 1 study both 3DCRT and IMRT for patients’ treatment (Table 1). The calculated biologically effective dose (BED) with α/β = 10 Gy (for acute toxicity) in all studies revealed that it was lower for the HF compared to the SF arm. It varied from 78 to 86.56 Gy in HF with 3–3.4 Gy/fraction versus 93.6 Gy in SF groups with generally 78/39 × 2 Gy schedules (Table 2).

### Scoring systems

From the involved studies, 10/17 reported GI toxicities using the EORT-RTOG scoring system, 1/17 used EORT-RTOG and CTCAE, and 6/17 studies used the CTCAE toxicity grading system (Table 2 & Table 3). Physician reported toxicity records were collected in all studies, but patient reported outcomes were presented in only 3/17 studies [2], [6], [20]. Available data revealed that patients’ symptoms peaked sooner in HF than in SF during the acute phase of radiotherapy. Late toxicity was reported using mainly EORT-RTOG; in some studies LENT-SOMA was used as well (Table 2 & Table 3).

#### Acute GI toxicity patterns

Reported grade ≥ 2 GI adverse events rates from involved studies are summarized in Table 2, and the study specific risk differences of acute grade ≥ 2 toxicities between HF and SF are summarized in Table 4 in Appendix A. In general, 2 trials [2], [4] recorded significant higher acute grade ≥ 2 GI toxicity rates in the HF arm. During radiotherapy, Aluwini et al. reported that 122/391(31.2 %) available patients in SF group and 169/403 (41.9 %) in HF side developed acute grade ≥ 2 GI (risk difference = 10.7 %, *p* = 0.0013). Dearnaley et al. recorded 176/715(24.6 %) acute grade ≥ 2 GI adverse events in SF, 277/720 (38.4 %) in one HF (60/20 × 3) (*p* < 0.0001) and 270/713 (37.8 %) in another (57/19 × 3) (risk difference = 13.8 %, *p* < 0.0001). Other studies did not report any significant differences in the recorded acute GI adverse events between HF and SF (Table 2).

A meta-analysis was done for the involved cohorts. In general, pooled results showed that the risk of acute grade ≥ 2 GI toxicity for moderate HF was increased by 6.3 % (95 % CI = 2 % to 10.6 %), I^2^ = 69.4 % (95 % CI = 29.1 % to 82.5 %) (Fig. 2). Chi^2^ (test risk difference differs from 0) = 8.2 (df = 1) *p* = 0.0041. Bias indicators: Begg-Mazumdar: Kendall's tau = −0.02, *p* = 0.86 (low power) and Egger bias = 0.35 (95 % CI = −22 to 29 %) *p* = 0.75 (Fig. 7, supplementary file).

### Acute GU toxicity patterns

Reported grade ≥ 2 GU adverse event proportions from involved studies are summarized in Table 3 and the study specific risk differences of acute grade ≥ 2 between HF and SF are summarized in Table 3. In general, the involved studies did not report significant differences in acute grade ≥ 2 GU toxicity rates between HF and SF arms during radiotherapy. The HYPRO trial reported 226/391 (57.8 %) acute grade ≥ 2 GU toxicity events in the SF group and 244/403 (60.5 %) in the HF group (risk difference = 2.7 %, *p* = 0.43) (2). The CHHiP trial recorded 331 /715 (46.2 %) acute grade ≥ 2 GU adverse events in the SF arm, 365/720 (50.6 %) in one HF arm (60/20 × 3) (risk difference = 4.4 %, *p* = 0.095) and 327/713 (45.8 %) in the second HF arm (57/19 × 3) (risk difference = 0.4 %, *p* = 0.87) [4]. Arcangeli et al. reported 34/85 (41 %) acute grade ≥ 2 GU events for SF and 39/83(46.9 %) for HF (risk difference = 5.9 %, *p* = 0.44) [7].

The meta-analysis of acute grade ≥ 2 GU toxicity revealed that the risk of acute grade ≥ 2 GU toxicity in moderate HF schedule was increased by 1.3 % (95 % CI = -10.9 % to 38.4 %), I^2^ = 0 % (95 % CI = 0 % to 52.7 %), which was not significant (Fig. 3). Chi^2^ (test risk difference differs from 0) = 1.19 (df = 1) *p* = 0.274 and Bias indicators: Begg-Mazumdar: Kendall's tau = −0.022 (*p* = 0.86 low power), Egger: bias = -0.177 (95 % CI = −13 % to 97 %) *p* = 0.73 (Fig. 8, appendix A).

#### Late GI toxicity patterns

Reported late grade ≥ 2 GI adverse events proportions from involved studies are summarized in Table 2, and the study specific RD of late grade ≥ 2 between HF and SF are summarized in (Table 5, appendix A).

A meta-analysis was done for the involved cohorts. In general, pooled results showed that the risk of late grade ≥ 2 GI toxicity in moderate HF schedule was increased by 0.23 % (95 % CI = -3.6 % to 4.1 %), *p* = 0.9, I^2^ = 80.18 % (95 % CI = 59.66 % to 90.26 %) (Fig. 4).

#### Late GU toxicity patterns

Reported late grade ≥ 2 GU adverse events proportions from involved studies are summarized in Table 3, and the study specific RD of late grade ≥ 2 between HF and SF are summarized in (Table 5, Appendix A). A meta-analysis was done for the involved cohorts. In general, pooled results showed that the risk of late G ≥ 2 GU toxicity in moderate HF schedule was increased by 1.84 % (95 % CI = −0.6 % to 4.3 %), *p* = 0.15, I^2^ = 42.55 % (95 % CI = 0.0 % to 78.85 %) (Fig. 5).

### Risk of bias analysis

We assessed risk of bias in involved studies by ROB2 Cochrane tool for RCT and a summary of assessment is presented by traffic light in Fig. 4 (Appendix A) and for observational comparative studies we used Newcastle-Ottawa risk of bias tool and is summarized results are summarized in Appendix D.

## Discussion

The current evidence-based guidelines paper that was published by ASTRO, ASCO and AUA expert’s consensus, strongly recommend the use of moderate HF (2.4–3.4 Gy/fraction) for localized prostate cancer patients who choose EBRT as their treatment modality. Moreover, it stated that there was no difference in acute GI and GU toxicity between patients treated with HF and those treated with SF radiotherapy but suggested that men being treated with moderate HF should be counselled about the slightly increased risk of developing acute GI toxicity [20].

In the current study, the meta-analysis included n = 10 studies that reported acute rectal and bladder complications caused by radiotherapy for patients treated with moderate HF or SF radiotherapy. In general, pooled results showed that the risk of acute grade ≥ 2 GI toxicity in moderate HF schedule was significantly increased by 6.3 % whereas acute GU toxicity showed no significantly increased risk with a point estimate of +1.3 %. Our findings are consistent with findings from Francolini et al. [13] who performed a meta-analysis for moderate HF with 3–4.5 Gy per fraction, evaluating acute toxicity, and reported a pooled risk difference (HF vs SF) of +9.8 % (95 % CI 4.8–14.7) for acute GI toxicity and no significant risk differences for acute GU toxicity (point estimate of +1.5 %). In a meta-analysis conducted by Baccaglini et al. [16] acute and late toxicity levels were compared between ultrahypofractionation groups (≥5 Gy fraction size) vs standard to moderate hypofractionation groups. They reported no significant risk differences for GU and GI toxicity. They did however not report separately on moderate vs standard hypofractionation, therefore a valid comparison with our results is not possible.

From the pooled results we observed an increased risk of 6.3 % for acute GI toxicity for patients treated with moderate hypofractionation schedules, in contrary to the calculated BED prediction. An increase of 6 % (or higher) was observed for 5 out of the 10 studies, and for most studies observing smaller risk differences, the 95 % confidence interval was overlapping with the pooled result of a 6 % risk difference, except for the study of Lee et al. [3], who observed very similar acute GI toxicity risks for HF and SF, with a 95 % confidence interval of −3.4 %–+3.8 % for the risk difference (Fig. 2). In this study of Lee et al. [3], the fraction size for HF was 2.5 Gy, while for all other studies the fraction size was at least 3 Gy which might explain this observed outlier.

Considering acute GU toxicity, our meta-analysis indicated no increased risks in patients treated with moderate HF. These findings are considered reliable since the reported heterogeneity value was low. Furthermore, this result was consistent with the findings of Francolini et al. [13]. In a secondary analysis of the HYPRO data evaluating patient-reported symptoms, we did however observe a significant increase of the patient-reported symptom of acute urinary straining [34]. One of the weak points in testing numerous patient-reported symptoms for significant differences is the risk of false-positive results because of the multiple testing, therefore it would have been interesting to compare these results with other studies. However, for moderate hypofractionation there is also no other study reporting on acute patient-reported symptoms, so this could not be evaluated in this meta-analysis.

With respect to acute toxicity risks it is important to realize that acute symptoms are temporary and typically resolve within 3 months after radiotherapy [2], [4]. On the other hand, there have been reports in literature that patients experiencing acute toxicity are at increased risk for late toxicity. This phenomenon of late toxicity occurring as a consequential effect of acute injury after radiotherapy for prostate cancer has been confirmed in a systematic review by Peach et al. [33]. However, in both the HYPRO trial and the CHHIP trial, the observed increased acute GI toxicity levels were not associated with increased late GI toxicity levels [4], [6].

The evaluated ten studies in this meta-analysis were assessed on their risk of bias (Table 6, Appendix A). None of the studies used had low risk of bias since all studies scored at least “some concern” in the risk domain regarding bias in measurement of the outcome. The reason for this is that in all these radiotherapy studies, it was not a “double-blinded” design, i.e. patients, treating physicians, and researchers knew the allotted treatment arm of a patient which is considered as a potential risk for biased toxicity scoring according to the applied criteria. However, as true double blinded studies in radiotherapy are very difficult to set-up, these studies should be considered as the best obtainable design within radiotherapy.

With respect to our secondary endpoint of late GI/GU toxicity, we observed similar toxicity levels for HF and SF which is consistent with previously reports from meta-analyses and review studies [14], [15], [16], [17]. Carvalho et al. [14] conducted a meta-analysis in 9 studies with 7317 patients. He reported that late GI toxicity was statistically the same between HF and SF (12.9 HF vs 16.2 % SF; RD − 0.01; 95 % CI; −0.04,0.02; *p* = 0.41; *I*^2^ = 58 %).There was no difference in late GU toxicity between the two schedules (28.7 HF vs 28.0 % SF; RD −0.01; 95 % CI; −0.04,0.03; *p* = 0.67; *I*^2^ = 52 %). Botrel et al. [15] conducted a meta-analysis in nine trials comprising 2702 patients. This study reported that the incidence of late GI and GU events was the same in HF and SF (late GI toxicity, RR 1.17, 95 % CI 0.79–1.72, P = 0.44; and late GU, RR 1.16, 95 % CI 0.80–1.68, P = 0.44). Baccaglini et al. [16] conducted a meta-analysis in 8 studies including 2929 patients with localized prostate cancer. Pooled analysis revealed no difference between late GI and GU adverse events (GI, 2.1 % HF × 3.5 %SF, RD − 0.01; 95 % CI −0.03, 0.00; p = 0.05; I^2^ = 22 % and GU, 3.9 % HF × 4.7 % SF, RD − 0.01; 95 % CI −0.03, 0.00; p = 0.16; I^2^ = 19 %). Yin et al. [17], conducted a meta-analysis on seven studies with 8,156 participants. Results revealed no significant difference in late gastrointestinal (RR = 0.97, 95 % CI: 0.71–1.33, *P* = 0.85) and genitourinary (GU) toxicities (RR = 1.04, 95 % CI: 0.87–1.24, *P* = 0.69) between HF and SF.

According to our findings, moderate HF is safe but also associated with a slight increase of acute GI sides effects, which is in agreement with the results of other review studies. Therefore, with the current limited global radiotherapy resources, HF is a good option to help patients from low-income countries, and increase the numbers that can be irradiated. However, one should keep in mind that with respect to toxicity risks, other relevant factors have to be taken into account as well such as differences in the radiotherapy techniques used, different dose levels, different target volumes, and differences in tumor stages, and patient positioning procedures with or without advanced imaging equipment. Furthermore, it remains crucial to obtain more information on how acute toxicity has to be handled optimally in a low-income country with respect to e.g. needed medication and preferred follow-up by the radiotherapist, urologist, and/or general practitioner. Therefore, further research is needed to understand more on the acute period of radiotherapy using hypofractionation and the required clinical management especially in countries where advanced intensity-modulated techniques and advanced imaging equipment is available.

## Conclusion

In conclusion, reports on the details of acute toxicity and its clinical management were limited. A significant increase of acute GI toxicity risk was observed for HF compared to SF, with an estimated risk difference of +6 %, needing adequate monitoring and management. Pooled late GI and GU toxicity showed similar levels with SF and HF.

## Declaration of Competing Interest

The authors declare that they have no known competing financial interests or personal relationships that could have appeared to influence the work reported in this paper.
