# Supplementary material for: Acute and late toxicity patterns of moderate hypo-fractionated radiotherapy for prostate cancer: A systematic review and meta-analysis
Source: Clin Transl Radiat Oncol. 2023 Mar 17;40:100612. doi: 10.1016/j.ctro.2023.100612 (PMC10040508; doi:10.1016/j.ctro.2023.100612)
Supplement: Supplementary data 1 [file mmc1.docx]

**Supplementary Files**

**Table 4**. Study specific G≥2 GI and GU acute toxicity risk difference (RD) between HF and SF with corresponding 95% Confidence Intervals (CI)

| **STUDY** | **Risk Difference GU** | **95% CI (Miettinen)** | **Weight %(fixed)** | **Risk Difference**  **GI** | **95% CI (Miettinen)** | **Weight% (Random)** |
| --- | --- | --- | --- | --- | --- | --- |
| Aluwini et al | 2.2% | -4.0% - 9.5% | 14.49 | 10.7% | 4.0 % -17.3% | 12.6% |
| Dearnaley et al | 4.4% | -0.7% - 95.5% | 26.19 | 13.8% | 9.0 % - 18.5% | 14.8% |
| Arcangeli et al | 6.9% | -80% - 21.6% | 3.07 | 13.7% | 0.1 % - 27% | 6.5% |
| Catton et al | -0.1% | -5.0% - 48.7% | 22.01 | 5.7% | 2.0 % - 9.5% | 15.9% |
| Karklelyte et al | -1.3% | -13.2% - 10.4% | 4.03 | 1% | 3.0 % - 22.8% | 6.8% |
| Viani et al | 1.6% | -8.8% - 11.9% | 3.96 | 1.6% | -8.8 -11.9% | 9.0% |
| Norkus et al | 0.6% | -17.5% - 18.9% | 2.04 | 0% | -13.2- 13.4 | 7.1% |
| Lee et al | -1% | -6.1% - 4.0% | 19.69 | 0% | -3.4% - 3.7% | 16.1% |
| Kozuka et al | -2.7% | -22.5% - 17.3% | 1.66 | 0.3% | -10.9% - 21.4% | 5.2% |
| Mc Donald et al | -4.7% | -20.1% - 10.8% | 2.86 | 0% | -14.2% - 15.6% | 5.6% |

Abbreviations: GI (Gastro-intestinal); GU (Genito-urinary); RD (Risk Difference); HF (Hypofractionation); SF (Standard Fractionation); CI (Confidence Interval)

**Table 5**. Study specific and cumulative G≥2 GI and GU late toxicity risk difference (RD) between HF and SF with corresponding 95% Confidence Intervals (CI)

| **STUDY** | **Risk Difference GU** | **95% CI (Miettinen)** | **Weight %(Radom)** | **Risk Difference**  **GI** | **95% CI (Miettinen)** | **Weight% (Random)** |
| --- | --- | --- | --- | --- | --- | --- |
| Aluwini et al | 2.25% | -0.046 – 0.091 | 10.02 | 4.97% | -0.005 - 0.105 | 15.04 |
| Dearnaley et al | 0.36% | -0.007 – 0.014 | 36.57 | -2.01% | -0.035 – 0.005 | 20.93 |
| Arcangeli et al | 2.55% | -0.052 – 0.103 | 8.25 | 2.69% | -0.075 – 0.129 | 8.72 |
| Catton et al | 0.83% | -0.036 – 0.053 | 17.44 | -3.64% | -0.069 – 0.003 | 18.76 |
| Lee et al | 5.64% | 0.006 – 0.107 | 15.32 | 6.74% | 0.025 – 0.110 | 17.25 |
| Kozuka et al | -1.31% | -0.165 – 0.139 | 2.56 | -0.02% | -0.075 – 0.070 | 12.6 |
| Mc Donald et al | 3.01% | -0.039 - | 9.84 | -11.1% | -0.231 – 0.010 | 7.05 |
| Cumulative | 1.84% | -0.006 – 0.043 | 100 | 0.23% | -0.036 – 0.041 | 100 |

**Figure 6**. Risk of bias score of included RCTs based on Cochrane Risk of Bias tool (RoB2). D = domain; D1: Bias arising from the randomization process; D2: Bias due to deviations from intended intervention; D3: Bias due to missing outcome data; D4: Bias in measurement of the outcome; D5: Bias in selection of the reported result.

|  | D1 | D2 | D3 | D4 | D5 | ***Overall*** |
| --- | --- | --- | --- | --- | --- | --- |
| *Aluwini et al. 2015* | Low | Low | Low | Some concerns | Low | ***Some concerns*** |
| *Dearnaley et al. 2016* | Low | Low | Low | Some concerns | Low | ***Some concerns*** |
| *Alcangeli et al. 2011* | Low | Low | Low | Some concerns | Low | ***Some concerns*** |
| *Clatton et al. 2017* | Low | Low | Low | Some concerns | Low | ***Some concerns*** |
| *Karklelyte et al. 2016* | High | Low | Low | Some concerns | Low | ***High*** |
| *Viani et al. 2013* | High | Some concerns | Low | High | Low | ***High*** |
| *Norkus et al. 2013* | Low | Low | Low | Some concerns | Low | ***Some concerns*** |
| *Lee et al. 2016* | Low | Low | Low | Some Concerns | Low | ***Some concerns*** |

**Figure 7**. Overall bias assessment for GI toxicity meta-analysis

**Figure 8**. Overall bias assessment for GU toxicity meta-analysis
